# Correlation of histopathological patterns of OSCC patients with tumor site and habits

**DOI:** 10.1186/s12903-022-02336-6

**Published:** 2022-07-23

**Authors:** Madiha Muhammad Yasin, Zia Abbas, Abdul Hafeez

**Affiliations:** Dow University of Health Sciences, Karachi, Pakistan

**Keywords:** Histopathological pattern, Tumor, OSCC, Habits, Oral cancer

## Abstract

**Introduction:**

Oral cancer is considered a major global public health problem. The causes of OSCC are tobacco, alcohol, viral infections such as EBV, HPV, and herpes simplex virus, poor oral hygiene (including sharp teeth and decay), ill-fitting denture, ultraviolet (UV) exposure, nutrition, and genetic predisposition. The etiology of oral cancer varies in different populations due to area-specific etiological factors.

**Objective:**

Finding a correlation of histopathological pattern to the tumor site and habits as an outcome of OSCC.

**Methods:**

This cross-sectional study was conducted in Karachi, Pakistan. A total of 100 known cases of an oral squamous cell carcinoma were diagnosed with the help of biopsy reports and were examined for histopathologic features, site of the lesion, and risk habits.

**Results:**

48 years was the mean age at the time of diagnosis with a distribution of 61% men and 39% women. The frequently affected site was buccal mucosa and the prime risk habit was gutka followed by betel quid. Histologically, the degree of differentiation shows that moderately differentiated OSCC was most commonly present, while the most prevalent histopathological pattern was spindle cell carcinoma. The statistical relation between lesion site and tobacco habits was found to be significant with a *p* value (*p* = 0.01).

**Conclusion:**

Rates of oral squamous cell carcinoma are higher in males than females with a mean age at the time of diagnosis being less than 50 years. Frequently placing gutka in the buccal vestibule against buccal mucosa is responsible to make buccal mucosa the most common tumor site. This study provides baseline information regarding habits.

**Supplementary Information:**

The online version contains supplementary material available at 10.1186/s12903-022-02336-6.

## Key points

- Gives an overview of the histopathological patterns of oral squamous cell carcinoma.
- Provides baseline dental registry data of OSCC patients, which associates their risk habits and site of the lesion with age and gender.
- This study suggests that habits within the Asian population play an important role in oral cancer incidence and clinical presentation.

## Introduction

Oral squamous cell carcinoma is the most frequently occurring carcinoma of the head and neck. Every year 2.5% of all newly reported carcinoma cases are of OSCC while the death rate is 1.9% [1].

OSCC arises from the mucous membrane of the oral cavity and may affect the buccal and labial mucosa, gingiva, retromolar area, floor of the mouth, hard palate, lip, mobile portions of the tongue, and cheeks [2].

The significantly associated risk factors of OSCC are tobacco, alcohol, viral infections such as EBV, HPV, and herpes simplex virus, poor oral hygiene (including sharp teeth and decay), ill-fitting denture, ultraviolet (UV) exposure, nutrition, and genetic predisposition. The etiology of oral cancer varies in different populations due to area-specific etiological factors [3].

Tobacco consumption in various forms, including areca nut, betel quid, beeri, gutka (mawa/khara), mainpuri, khaini (tobacco and lime), khiwam, naswar, zarda (boiled tobacco), supari, pan, and an attractive sachet of pan masala is an important etiological factor for developing OSCC [3].

The eating habits of areca nut, pan, and betel quid are mostly found in South Asian countries and correlate with tumor sites as well [4]. Their easy accessibility in economical, appealing sachets gets them common among all ages, involving children, teenagers, and adults [4].

The chemical composition of betel quid comprises carbohydrates, proteins, fats, alkaloids, coarse fiber, and minerals such as sodium, calcium, manganese, and copper. Four alkaloids discovered in betel quid involve arecoline, arecaidine, guvacine, and guvacoline [5].

Alcohol plays an important role as a major risk component in oral carcinoma. Alcohol consumption is more common in Europe and North America. The known proportion of severe alcoholic is relatively less in South Asian countries, it is also assumed that most of the alcoholic drinks used in these populations are not proven by documents [6].

The treatment and prognosis of OSCC are based on the tumor site and the histological degree of differentiation [7]. Risk habits of tobacco consumption contribute to differential behaviors of risk factors attributed towards different anatomic zone frequencies [8].

Histologically, there are different types of OSCC. Conventional oral squamous cell carcinoma can present as several variants that make up an aggregate of about 10–15% of all squamous cell carcinomas (SCC). These variants involve verrucous carcinoma (VC), adenoid/acantholytic/pseudoglandular SCC (AdSCC), spindle cell/sarcomatoid carcinoma (SCSC), adenosquamous carcinoma (ASC), papillary SCC (PSCC) and basaloid SCC (BSCC). Each of the variants has a different histomorphological pattern. The verrucous carcinoma is a well differentiated low grade malignant variant of SCC as compared to basaloid, a more aggressive form of SCC [9].

Differentiation is the degree to which deformed cells look like usual normal cells. The degree of differentiation represents grading of OSCC which includes well differentiated, moderately differentiated, and poorly differentiated carcinoma [10].

Early diagnosis and treatment are goals. Biopsies are believed to be the gold standard for lesion identification. A biopsy may be incisional (wedge or punch) or excisional, and is necessary to confirm the diagnosis [10].

Several studies have been acknowledged for the rising incidence of OSCC over decades, but the correlation of histopathological patterns to lesion sites and tobacco habits is still limited. This study is helpful to provide current knowledge regarding histopathological patterns and habits associated with OSCC, but our limited data shows the result of patients related to only this hospital, not the whole region.

## Materials and methods

### Research design and settings

This cross-sectional study was conducted in Karachi, Pakistan. A total of 100 known cases of an oral squamous cell carcinoma between 2019 and 2021 were included in the OMF department at Dow University of Health Sciences. The cases were diagnosed with the help of biopsy reports and has been examined for histopathologic features, site of the lesion, and risk habits.

The biopsy forms contained information such as patient demographics (patient name, gender, date of birth), risk factors, quality of life, previous history, and clinical features of the lesion biopsied.

Patient oral or written informed consent was obtained before starting the interview. For this purpose, confidentiality was assured. An audio recording was kept confidential only by the researcher and was discarded as the study ended.

Before data collection, all participants had received a patient information sheet, a verbal description of the cause of the study, and a questionnaire. They had invited to ask questions for clarification at the end of the explanation. They had informed that all completed questionnaires will offer complete anonymity to the participant and the data collected would be treated with confidentiality.

Age and gender were included as independent variables while risk habits, anatomical site of lesion, biopsy technique, histologic type, and histologic grading were dependent variables.

Inclusion criteria include patients with known cases of OSCC with a mouth opening of more than 20–25 mm, age above 18 years, patients who are aware of the diagnosis, and who can understand and answer the questionnaire on their own or with an explanation of investigators.

Exclusion criteria consist of patients not willing for biopsy, patients with limited mouth opening, less than 20 mm. Patients who had received chemotherapy or radiotherapy, and patients with recurrent diseases.

### Data collection and analysis

The primary data gathering approach was followed for this research study. It mainly comprised questionnaires, interviews, observations, and biopsy reports. In addition, all patients with known cases of OSCC were part of this study.

Patients were provided a ‘Patient Information Sheet’ and consent was sought on the ‘Consent Form’.

The software used for all statistical analyses was IBM SPSS Base Licensed version 23. Categorical data were analyzed using Pearson’s chi-squared test. Odds proportion and subsequently 95% confidence intervals were analyzed. Each test was two-sided and the results were considered statistically significant when *p* < 0.05.

A cross table was made to see the association between the pattern of OSCC with tumor site and habits by using the Chi-square test. A *p* value of 0.05 or lower will be considered significant.

## Results

The mean age at diagnosis was 48 years. Of which 63% were males and 37% were female. The association of gender with tumor site was determined through Pearson’s chi-squared test. Statistically, the distribution of tumor sites concerning gender is found to be significant with a *p* value of 0.04.

The buccal mucosa was the most affected site in males (73.8%), while tongue involvement was higher in females (60.0%) as shown in Table 1.

**Table 1:** Demographic distribution of gender with lesion site

lesion site	Gender		*P* value
	Male	Female	
Buccal mucosa	31 (73.8%)	11 (26.2%)	0.04
Lower gingiva	10 (62.5%)	6 (37.5%)	
Tongue	8 (40.0%)	12 (60.0%)	
Retromolar trigone	5 (62.5%)	3 (37.5%)	
Lip	4 (100%)	0	
Alveolar ridge	1 (25.0%)	3 (75.0%)	
Hard palate	1 (33.3%)	2 (66.7%)	
Floor of mouth	3 (100%)	0	
Total	63 (63.0%)	37 (37.0%)	

In the current study, 14 (13.7%) respondents committed that they were involved in multiple habits of tobacco consumption, which not only consist of gutka, naswar, betel quid, pan, and smoking but also include different types of supari, mawa, mainpuri and pan masala.

**Fig. 1:**
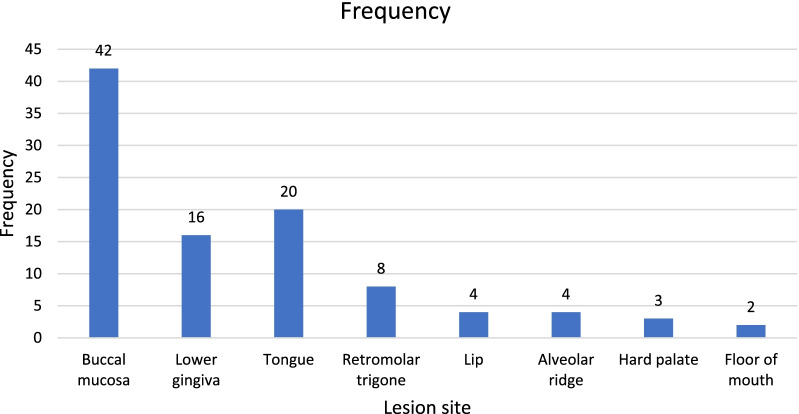
Distribution of anatomical lesion site of OSCC

In smokeless tobacco, gutka (17.6%) is the most common chewing habit. Next to gutka, the most commonly used tobacco is betel quid (16.7%) and naswar (14.7%) followed by pan (12.7%), smoking (5.9%), and alcohol (1.0%). Around 16 (15.7%) of patients have never been involved in any kind of tobacco consumption but still suffering from OSCC. The frequency of oral squamous cell carcinoma, according to different types of tobacco consumption is shown in Table 2 and Fig. 1.

**Table 2:** Distribution of Lesion site with tobacco habits

Lesion site	Tobacco habit								*P* value
	Multiple habits n (%)	Gutka n (%)	Naswar n (%)	Betel quid n (%)	Pan n (%)	Smoking n (%)	Alcoholn (%)	No habits n (%)	
Buccal mucosa	8 (19.0)	15 (35.7)	7 (16.7)	6 (14.3)	4 (9.5)	0	0	2 (4.8)	0.01
Lower gingiva	3 (18.8)	2 (12.5)	1 (6.3)	3 (18.8)	3 (18.8)	2 (12.5)	0	2 (12.5)	
Tongue	1(5.0)	0	2 (10.0)	5 (25.0)	2 (10.0)	1 (5.0)	1 (5.0)	8 (40.0)	
R.* trigone	0	0	4 (50.0)	1 (12.5)	2 (25.0)	0	0	1 (12.5)	
Lip	0	0	0	0	1 (25.0)	2 (50.0)	0	1 (25.0)	
Alveolar ridge	1 (25.0)	1 (25.0)	0	1 (25.0)	1 (25.0)	0	0	0	
Hard palate	0	0	0	1 (33.3)	0	1 (33.3)	0	1 (33.3)	
Floor of mouth	1 (33.3)	0	1 (33.3)	0	0	0	0	1 (33.3)	
Total	14 (14.0)	18 (18.0)	15(15.0)	17(17.0)	13(13.0)	6(6.0)	1 (1.0)	16(16.0)	

*R = Retromolar

The statistical relation between lesion site and tobacco habits was found to be significant with a *p* value (*p* = 0.01).

In this study, the very frequent anatomical location with OSCC was buccal mucosa (42.2%) followed by lower gingiva (15.7%), tongue (19.6%), retromolar trigone (7.8%), lip and alveolar ridge (3.9%), hard palate (2.9%) and floor of mouth (2.0%) as shown in Fig. 1.

**Fig. 2:**
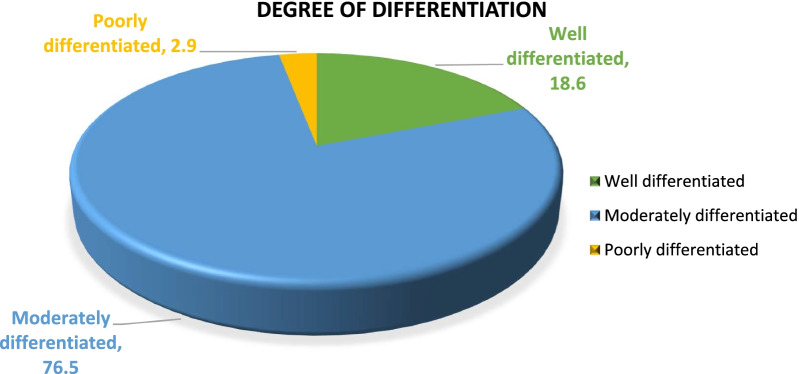
Degree of differentiation of OSCC

According to histological grades, the degree of differentiation shows that moderately differentiated oral squamous cell carcinoma was most commonly present in 78 (76.5%) followed by well-differentiated 19 (18.6%) and poorly differentiated 3 (2.9%) as shown in Fig. 2.

The correlation of histopathological pattern to lesion site and habits is determined through Pearson’s chi-squared test, have a *p* value of 0.04 is found to be statistically significant as shown in Table 3. The level of significance for this study is 0.05. The SPSS version 23 was applied to carry out statistical analysis.

**Table 3:** Correlation of histopathological patterns to lesion site and habits

Factor	Histological patterns					*P* value
	Spindle cell n (%)	Verrucous n (%)	Basaloid n (%)	Papillary n (%)	Mucoepidermoid n (%)	
**Lesion site**						
Buccal mucosa	18 (42.9)	20 (47.6)	4 (9.5)	0 (0.0)	0 (0.0)	0.047
Lower gingiva	6 (37.5)	8 (50.0)	0 (0.0)	1 (6.3)	1 (6.3)	
Tongue	16 (80.0)	1 (5.0)	0 (0.0)	3 (15.0)	0 (0.0)	
Retro molar trigone	4 (50.0)	3 (37.5)	0 (0.0)	0 (0.0)	1 (12.5)	
Lip	2 (50.0)	1 (25.0)	1 (25.0)	0 (0.0)	0 (0.0)	
Alveolar ridge	2 (50.0)	1 (25.0)	0 (0.0)	1 (25.0)	0 (0.0)	
Hard palate	2 (66.7)	1 (33.3)	0 (0.0)	0 (0.0)	0 (0.0)	
Floor of mouth	2 (66.7)	0 (0.0)	1 (33.0)	0 (0.0)	0 (0.0)	
**Risk habits**						
Multiple habits	1 (7.1)	10 (71.4)	2 (14.3)	1 (7.1)	0 (0.0)	0.046
Gutka	8 (44.4)	8 (44.4)	2 (11.1)	0 (0.0)	0 (0.0)	
Naswar	11 (73.3)	3 (20.0)	0 (0.0)	0 (0.0)	1 (6.7)	
Betel quid	8 (47.1)	7 (41.2)	1 (5.9)	1 (5.9)	0 (0.0)	
Pan	8 (61.5)	3 (23.1)	0 (0.0)	2 (15.4)	0 (0.0)	
Smoking	2 (33.3)	2 (33.3)	1 (16.7)	0 (0.0)	1 (16.7)	
Alcohol	1 (100)	0 (0.0)	0 (0.0)	0 (0.0)	0 (0.0)	
No habits	13 (81.3)	2 (12.5)	0 (0.0)	1 (6.3)	0 (0.0)	

The most prevalent histopathological pattern is spindle cell carcinoma (52%) followed by verrucous cell carcinoma (35%) as shown in Table 4. Six percent (6%) of patients have more aggressive basaloid cell carcinoma. Five (5%) of patients have papillary cell carcinoma and 2% mucoepidermoid.

**Table 4:** Histological subtypes of OSC identified in participants

Histologic type	Percent
Spindle cell carcinoma	52
Verrucous cell carcinoma	35
Basaloid cell carcinoma	6
Papillary cell carcinoma	5
Mucoepidermoid	2
Adenoid squamous cell carcinoma	0

## Discussion

Oral squamous cell carcinoma is the most common malignant neoplasm, derived from the mucosal epithelium in the oral cavity, responsible for confined injurious growth and distant metastasis [16].

In the present study, patients demonstrated ages ranging from 28 to 75 years, in which most of the cases were seen in the fifth decade. The mean age of patients reported in our study is 48.33, according to the results of other several South Asian studies [17–19], yet in contrast to findings from the European studies [1, 22, 23], where the mean age at diagnosis was 63 years. This indicates that there is a rising incidence of the younger age group in South Asia as there is greater exposure to risk habits.

Results from gender findings show that the majority were males, which was similar to local studies [17, 18] as well as international studies [1, 20].

This finding suggests that males belonging to the South Asian countries are at an increased risk of oral cancer as most of them are blue-collar workers and to kill the hunger strike, they consume different forms of tobacco products in their daily life.

The results are different when lesion site is concerned, the study conducted in Lahore by Rakia et al. [19] and Abdul et al. [8] reports the tongue as the most frequently involved site while the study carried out in Copenhagen by Schmidt Jensen et al. [1] reports floor of the mouth. According to our study, the most common anatomical sublocation was buccal mucosa (42.2%) followed by lower gingiva (17.6%). This variation could be due to the exposure of risk habits such as gutka which is the most used smokeless tobacco in our study. The users generally hold gutka against the gum line for a long period, as the nicotine is absorbed, which affects buccal mucosa the most.

When the histopathological pattern is concerned, spindle cell carcinoma is most frequently occurred (52%) in our study. This result is almost similar to other studies as well [8]. Thirty-five percent (35%) of patients in the current study have a Verrucous form of well-differentiated squamous cell carcinoma, which is a lesser aggressive form compared to the more aggressive basaloid form comprises six percent (6%) of patients. While according to Neville et al. [21], 1–10% of all oral squamous cell carcinomas were verrucous carcinoma.

The reported cases of papillary squamous cell carcinoma were 5% in our study, which is most commonly present on the tongue with a mean age of 56 years. While Caltabiano et al. [22] revealed that papillary squamous cell carcinoma is a rare variant of squamous cell carcinomas and accounts for approximately 1% of all cases [22].

According to the histological grades, the degree of differentiation shows that moderately differentiated oral squamous cell carcinoma was most commonly present (76.5%) in our study followed by well-differentiated (18.6%) and poorly differentiated (2.9%). On the contrary, Ramasamy et al. [23] and Talat et al. [24] reported a high percentage of well-differentiated OSCC in their studies.

## Conclusion

Among the targeted population chewing tobacco habits with male predominance is concluded as a major factor of OSCC. Frequently placing gutka in the buccal vestibule against buccal mucosa is responsible to make buccal mucosa the most common tumor site. An association of histopathological patterns to the lesion site and tobacco habits is determined through chi-square and was found to be statistically significant as shown in Table 3. The prevalence of OSCC can be decreased by increasing oral cancer knowledge among the overall population regarding habits such as the toxic effects of tobacco chewing and smoking. This study also provides baseline registered data for further study.

## Supplementary Information

**Additional file 1.** Raw Data.

## Data Availability

All data is provided in the attached Additional file 1 name “OCSS-Data-file.xlsx”.
